# What do patients find most helpful in group treatment? Importance of group therapeutic factors in standardized psychological group treatments

**DOI:** 10.1192/j.eurpsy.2024.793

**Published:** 2024-08-27

**Authors:** A. Rodríguez-Rey, F. Piazza, X. Segú, A. Ruiz, C. Sorroche, G. Virgili, X. Torres, I. Morilla

**Affiliations:** ^1^Clinical Health Psychology Section, Psychiatry and Clinical Psychology Service; ^2^Collaboration Program with Primary Care, Psychiatry and Clinical Psychology Service, Hospital Clínic to Barcelona, Barcelona, Spain

## Abstract

**Introduction:**

There are numerous structured group psychological treatments (GPT), especially in the cognitive behavioral paradigm, which have proven effective. In these TPG, strategies, guidelines, knowledge, etc. are worked and, in many cases, homework is prescribed as an integral part of the treatment. A group context is also generated where people relate, generally with a similar culture, ages, mental health states and life problems

**Objectives:**

Elucidate which group therapeutic factors (GTF) are valued as most important by patients in their psychological improvement process. Know what our patients consider has helped them most in their GPT, whether the GTF or the content of the therapy (CT), conceptualized as the set of guidelines, knowledge, strategies, exercises and learning carried out with the therapists intrasession and with the material provided intersessions

**Methods:**

A total of 36 patients(mean age=51.04 (9.21)); 69.44% women (n=25); with main diagnoses (77.77%, n=28) of adaptive disorder, 6 patients of major depression (16.66%) and 2 unspecified anxiety disorders (5.55%) are included in GPT based on acceptance and commitment therapy (ACT) of Hayes’s (2012) for primary care patients, and on a treatment protocol developed in our clinical health psychology section (Segú et al. PaP 2023; 25 6-18) in long covid patients

Patients are recruited and cared for in the collaboration program with the primary care centers (CPPC), n=22(61.11%), and 12 patients (38.89%) diagnosed with long covid in the specialized post-covid unit of internal medicine, and treated in the clinical health psychology section on the Hospital Clínic of Barcelona (HCB)

Post-treatment evaluation is carried out using the GTF questionnaire, based on Yalom’s Q-short(1985), validated with 11 items, adapted to Spanish (Ribé et al. RAEN 2018; 38(134) 473-89). Patients rate from 1 to 10 how much they consider each FTG has helped them in their improvement process

**Results:**

The relevance of the GTF are: Altruism(8.16), catharsis(7.61), cohesiveness(7.94), corrective recapitulation(6.15); socialization techniques (6.41); self-awareness of reality(6.65); imitative behavior(6.43); participated information(6.69), instill hope(6.39); interpersonal learning (7.07), universality(8.27).

Regarding the other objective, 44.44%(n=16) consider the GTF more important than the content of the therapy in their improvement; 36.11%(n=13) equal importance; 13.88%(n=5) plus the CT and 2 consider that none of it has helped them (5.55%). Total importance CT(7.18/10) and GTF(7.44/10). The perceived help in their improvement process in the GPT(CT + GTF)=7.61/10.

**Conclusions:**

In two structured group treatments, based on ACT, a greater percentage of patients value that the GTFs have helped them more in their improvement process than the CT. The GTFs considered most relevant were universality, altruism, cohesiveness and catharsis.

**Disclosure of Interest:**

None Declared

